# 57437 Effects of Prebiotics on the Gut Microbiome Profile, Beta-cell Function and Immune Markers in Newly-Diagnosed Type 1 Diabetes

**DOI:** 10.1017/cts.2021.502

**Published:** 2021-03-30

**Authors:** Heba M. Ismail, Carmella Evans-Molina, Linda A. DiMeglio

**Affiliations:** Indiana University School of Medicine

## Abstract

ABSTRACT IMPACT: The proposed research study will provide critical pilot data on the effect of using the prebiotic (HAMS-AB) on the gut microbiome profile, Beta-cell function and immune markers in humans with T1D. OBJECTIVES/GOALS: The overall objective of this study is to assess how the prebiotic high amylose maize starch that has been acetylated and butyrylated (HAMS-AB) impacts the gut microbiome profile, short chain fatty acid (SCFA) production, glycemia, Beta-cell function/health and immune responses in newly diagnosed youth with type 1 diabetes (T1D). METHODS/STUDY POPULATION: We are performing a pilot randomized cross-over trial. We plan to recruit 12 newly-diagnosed T1D youth with residual Beta-cell function between 12-16 years of age. We will profile the gut microbiome using metagenomics, measure stool SCFA levels using mass spectrometry, assess glycemia using continuous glucose monitoring, assess insulin production using mixed meal tolerance testing, assess Beta-cell stress using proinsulin/C-peptide levels, and test immune responses by examining cytokine levels and frequency, phenotype and function of T cell markers in peripheral blood. RESULTS/ANTICIPATED RESULTS: Thus far, we have enrolled 3 participants, 1 has completed the study. Baseline assessments indicate that we have technical feasibility of performing the above studies and measurements. Recruitment and enrollment are ongoing. We hypothesize that the use of HAMS-AB in newly diagnosed youth with T1D will (i) improve the gut microbiome profile, (ii) increase SCFA production, (iii) improve overall glycemia and Beta-cell function and (iv) modulate the immune system and mitigate autoimmunity. DISCUSSION/SIGNIFICANCE OF FINDINGS: Given the failure to develop a cure for T1D despite multiple completed intervention studies and the unknown long-term effects of immune-modulatory therapy on those at risk for or those diagnosed with T1D, prebiotics such as HAMS-AB may offer a simple, safe, yet inexpensive and tolerated dietary alternative approach to mitigating disease.

